# Depressive Symptoms among People Living with HIV Attending ART Centers of Lumbini Province, Nepal: A Cross-Sectional Study

**DOI:** 10.1155/2023/3526208

**Published:** 2023-10-23

**Authors:** Saneep Shrestha, Upasana Shakya Shrestha, Jyoti Priyanka, Pragya Shrestha

**Affiliations:** ^1^Department of Community Medicine, Universal College of Medical Science and Teaching Hospital, Tribhuvan University, Bhairahawa, Rupandehi, Nepal; ^2^Care Nepal, USAID Adolescent Reproductive Health, Birendranagar, Surkhet, Nepal; ^3^School of Nursing and Midwifery, Karnali Academy of Health Sciences, Jumla, Nepal

## Abstract

**Background:**

Depression is a common mental disorder and is a leading cause of disability globally. Depressive symptoms among people living with HIV can be a significant barrier to ART initiation and thus lead to poor ART adherence. Global studies have found the prevalence of depressive symptoms among people living with HIV ranges from 12 to 63%. The real scenario of Nepal still needs to be explored. Thus, this study aimed to identify the prevalence and predictors of depression in individuals with HIV.

**Methods:**

An institutional-based cross-sectional study was carried out from August to December 2020 among 406 people living with HIV attending ART centers in Lumbini province. Participants were selected using a systematic random sampling technique and surveyed with a structured questionnaire consisting of sociodemographic variables, HIV AIDS-related variables, and 21 items Beck Depression Inventory tool. The odds ratio was used as the ultimate measure of association, with a 95% confidence interval computed to establish statistical significance. A multivariate regression analysis was carried out to identify the final predictors of depressive symptoms.

**Results:**

The study found that 26.8% of the respondents had depressive symptoms. Those who were literate (AOR = 0.24, 95% CI: 0.10–0.61), in the poorest wealth quintile (AOR = 7.28, 95% CI: 2.22-23.87), initiated ART within 12 months (AOR = 1.88, 95% CI: 1.03–3.42), had CD4 cell counts below 200 (AOR = 2.50, 95% CI: 1.54–4.06), and had a time difference of 3 months or less between HIV diagnosis and ART initiation (AOR = 0.50, 95% CI: 0.29–0.86) were independently associated with depressive symptoms.

**Conclusion:**

Routine screening for depressive symptoms should be integrated into national HIV prevention and control programs for people living with HIV. An enabling environment should be created to facilitate the rapid enrollment of individuals newly diagnosed with HIV in ART services, thereby reducing the time gap between HIV diagnosis and ART initiation.

## 1. Background

The World Health Organization (WHO) defines mental health as a state of wellbeing in which individuals recognize their potential, cope with the normal stresses of life, work productively, and contribute to their community [1]. Globally, the two most prevalent mental disorders are depression and anxiety, which ranked among the top 25 leading causes of burden worldwide in 2019 [2, 3].

Depression is a common mental disorder characterized by persistent sadness and a loss of interest in previously enjoyable activities [4]. The symptoms of depression can impact how a person feels, thinks, and handles their daily activities [5]. Depression is the leading cause of disability worldwide and a significant contributor to the overall global burden of disease [4]. The global prevalence of depression is estimated to be 3.4%, with variation across countries ranging from 2.9% in the Solomon Islands to 6.3% in Ukraine [6].

The Joint United Nation Program on HIV/AIDS (UNAIDS) 2021 report states that 38.4 million people are currently living with the human immunodeficiency virus (HIV) and 28.7 million are receiving antiretroviral therapy (ART), a significant increase from 7.8 million in 2010 [7]. There are currently 22375 people on ART service in Nepal, of which 4325 (19.32%) belong to Lumbini province, the second highest [8]. Although Nepal has made substantial progress in its HIV prevention program by reducing the HIV incidence per 1000 population from 0.08% in 2010 to 0.02 in 2021 and increasing the number of ART sites from 2 in 2004 to 84 in 2022 [8], the mental health dimension of people living with HIV (PLHIV) often goes unnoticed and unaddressed. The global prevalence of depression among PLHIV ranges from 6.73% to 89.9% [9, 10]. A recent meta-analysis study revealed the pooled prevalence of depression in PLHIV to be 31%, with the highest prevalence of 40% in the Southeast Asia region [11]. Studies in Nepal show it ranges from 25.5% to 43%, with occupational and marital status and ART duration as contributing factors [12, 13]. However, further exploration is needed to understand the scenario of depression and its associated factors among PLHIV in Nepal. Depression is a significant barrier to ART initiation and adherence among PLHIV in low-income countries [14–16]. Therefore, this study aims to determine the prevalence of depressive symptoms and associated factors among PLHIV. To our knowledge, this is the third study in Nepal and the first global study to assess the relationship between wealth quintile, time gap from HIV diagnosis to ART initiation, and depressive symptoms.

This study also aims to provide research evidence to other researchers, health care service providers, and policymakers for designing effective interventional programs to reduce depression prevalence, improve ART adherence, retention, achieve universal health coverage, and sustainable development goal (SDG) 3 where all the individuals will have good health and wellbeing.

## 2. Methods and Materials

### 2.1. Study Design and Setting

An institutional-based cross-sectional study was conducted from August to December 2020 at Lumbini Provincial Hospital, Prithivi Chandra Hospital, and Maharajgunj Primary Health Care Center in Lumbini Province, Nepal. Lumbini Province is in western Nepal and is the third-largest and most populous province. It covers an area of 22,228 sq. km, or 15.11% of the total country area.

### 2.2. Study Population

Participants in the study were HIV-positive individuals aged 18–60 years and enrolled in a selected ART center in Lumbini province. Patients who were grieving within six months, had not undergone a CD4 count in the six months prior to data collection, had known mental illness, and were female participants who were pregnant or in postpartum during the data collection period were excluded from the study.

### 2.3. Sample Size and Sampling Techniques

The lumbini province of Nepal consists of twelve districts, of which six are terai districts. There are eight ART centers in the Terai districts of Lumbini province, Nepal. ART centers in terai districts of Nepal were divided into three levels: provincial, district, and community. One center was selected from each level using the lottery method. The study identified eligible participants through the ART register and used a systematic random sampling method to select participants from each ART site. Participants were surveyed with a structured questionnaire containing socio-demographic variables, HIV-related variables, and the 21-item Beck depression inventory (BDI) tool. The required sample size was computed using the Stat Calc function of Epi-Info software.

(1) $$\begin{array}{c} N=\frac{Z^{2}*\text{pq}}{E^{2}}, \end{array}$$

where *Z* = standard variate, *E* = error term, *p* = prevalence of Depressive symptoms, and *E* is the allowable error.

A prevalence of 50% has been taken in this study to ensure a maximum sample size. From the above formula, taking *p* = 0.5, *q* = 1 − *p* = 0.5, and an allowable error of 5%, the sample size was *n* = 385. Considering a 5% nonresponse rate, the total sample was 406.

### 2.4. Data Collection Tool and Measurement

Data was collected using a structured questionnaire comprising sociodemographic variables, HIV-related variables, and a 21-item Beck Depression Inventory (BDI) tool. The study examined the sociodemographic characteristics of participants, including age, gender, marital status, educational status, employment status, and personal habits such as alcohol consumption and tobacco use. HIV-related variables included WHO clinical stage, duration of ART initiation, period since diagnosis, CD4 count, and time duration between HIV diagnosis and ART initiation. The WHO clinical stage of HIV has been classified into four stages. In clinical stage I, patients are asymptomatic or have persistent generalized lymphadenopathy for longer than 6 months, clinical stage II refers to a mildly asymptomatic stage characterized by unexplained weight loss of less than 10 percent body weight, recurrent respiratory infections, as well as the range of dermatological conditions [17]. Clinical stage III refers to a moderately symptomatic stage characterized by weight loss of more than 10 percent body weight, prolonged (more than one month) unexplained diarrhea, pulmonary tuberculosis, and severe bacterial infections [17]. Similarly, clinical stage IV refers to the severe symptomatic stage and includes all AIDS-defining illnesses [17]. Clinical variables of HIV-positive participants were collected from their ART register records. Beck depression inventory (BDI) scale was used to assess depressive symptoms. The BDI scale is composed of 21 items, each with a four-point scale ranging from 0 to 3. Therefore, the value of the BDI scale ranges from 0 (lowest) to 63 (highest). The Beck Depression Inventory (BDI) was used to assess depression severity, with scores ranging from normal (0–13), mild (14–19), moderate (20–28), and severely depressed (29–63). The tool has been validated in Nepal with a sensitivity of 0.92 and a specificity of 0.82 [18]. Also, the Cronbach alpha computed in our study was 0.948 which shows strong internal consistency and concurrent validity of the tool. ART counselors received a one-day orientation on data collection, and the collected data were regularly reviewed for errors by the principal investigator.

### 2.5. Data Processing and Analysis

Data were entered and analyzed in Statistical Package for Social Science (SPSS) software version 25. The mean and standard deviation were calculated to describe the characteristics of the sample. The bivariate logistic regression analysis assessed the association between dependent and independent variables. The ultimate measure of association was the odds ratio, and a 95% confidence interval was computed to determine statistical significance. Variables associated with bivariate analysis were entered into a multivariate logistic regression model to identify the final predictors of depressive symptoms among people living with HIV.

### 2.6. Ethical Consideration

Ethical approval was obtained from the Universal College of Medical Science and Teaching Hospital Institutional Review Committee (UCMS/IRC/014/20). Permission was obtained from the Medical Superintendent and Chief of the ART center of selected sites to access participants' clinical records. Participants were informed of the study, and written informed consent with a sign or thumbprint was obtained for data collection.

## 3. Results

### 3.1. Sociodemographic Characteristics of Study Participants

Table 1 shows the sociodemographic characteristics of study participants at selected ART centers in Lumbini province from August to December 2020. A total of 406 PLHIV participants participated in the study. The mean age of study participants was 40.71 years with a standard deviation of ±9.15 years. Among study participants, 162 (39.9%) were of age group 40–50 years, 207 (51%) were female, 122 (30%) were of religious minorities group, 280 (69%) were illiterate, 131 (32.3%) were engaged in the agriculture sector, 247 (60.8%) were living in a nuclear family, 258 (63.5%) were married, 295 (72.7%) and 298 (73.4%) did not consume tobacco and alcohol products three months preceding the survey, respectively.

### 3.2. Sociodemographic Characteristics of the Respondent's Partner

Table 2 shows the sociodemographic characteristics of the respondent's partner. The mean age of the respondent's partner was 39.49 years, with a standard deviation of ±7.75 years. Among respondent's partners, 106 (41.1%) were aged between 30−40 years, 179 (69.4%) were illiterate, 90 (34.9%) were involved in the agriculture sector, and 196 (76%) did not consume tobacco or alcohol products in the three months prior to the survey.

### 3.3. Depression and Clinical Characteristics of Study Participants

Table 3 shows the depression and clinical characteristics of study participants at selected ART centers in Lumbini province. Among the study participants, 109 (26.8%) had depressive symptoms. The majority of the study participants with a period of diagnosis of HIV more than 12 months from the survey date were 344 (84.7%). Similarly, 341 (84%) of the study participants were on ART medication for over 12 months. The time difference between HIV diagnosis and ART initiation with ≤3 months was 316 (77.8%). Half of the study participants, 205 (50.5%), were on WHO clinical stage one, and almost three-fourth, 301 (74.1%) of the study participants had a CD4 cell count of ≥200 cells/mm^3^.

### 3.4. Sociodemographic Predictors of Depression

Table 4 shows sociodemographic predictors of depression. Results from the bivariate analysis revealed that educational status and occupation of the respondent, family type, wealth quintile, and occupation of the respondent's partner were associated with depression. Factors significant in bivariate analysis were entered into the multivariate regression model for confounding adjustment. The multivariate regression model results identified educational status and wealth quintiles as sociodemographic predictors of depression. Literate study participants were found to have a protective effect on depression (AOR = 0.40, CI: 0.17–0.95) compared to illiterate participants. The odds of depression among the poorest wealth quintile were 7.28 times higher than the richest (AOR = 7.28, CI: 2.22–23.87).

### 3.5. Clinical Predictors of Depression

Table 5 shows the clinical predictors of depression. Factors associated with depression, resulting from the bivariate analysis, were entered into a multivariate model, which identified the period of ART initiation, the time difference between the period of diagnosis and the period of ART initiation, and CD4 cells count as clinical predictors of depression. The odds of depression among study participants with a period of ART initiation ≤12 months were 1.88 times higher than study participants with a period of diagnosis >12 months (AOR = 1.88, CI: 1.03–3.42). Similarly, the time difference between the period of diagnosis and ART initiation with ≤3 months was found to have a protective effect against depression (AOR = 0.50, CI: 0.29–0.86). The odds of depression were 2.50 times higher in study participants with a low CD4 cell count (AOR = 2.50, CI: 1.54–4.06).

## 4. Discussion

This study aimed to determine the prevalence of depressive symptoms and associated factors among PLHIV enrolled in ART centers in the Terai districts of Lumbini province, Nepal. The prevalence of depressive symptoms was 26.8% which is in line with the studies conducted in Nepal (25.9%) [13], Vietnam (26.2%) [19], Ethiopia (26.2%) [20], Cameroon (26.7%) [21], and Bhutan (27.2%) [22]. However, the prevalence of depressive symptoms in this study was lower than in previous studies conducted in Bangladesh (62.1%) [23], Pakistan (44%) [24], India (58.75%) [25], Sudan (63.1%) [26] and different states of Ethiopia, where the prevalence of depressive symptoms was found to be 41.7% [27], 35.5% [28], 32.6% [29], and 31% [30] respectively. Also, the prevalence of this study was higher than studies conducted in China (6.73%) [9], Guinea (16.9%) [31], Ethiopia (14.6%) [32], Vietnam (19.9%) [33], and Malawi (12%) [34]. The observed difference in prevalence rate might be due to different study populations, cultural settings, study time, sample size, and the use of different diagnostic tools to assess depressive symptoms.

The educational status of study participants was significantly associated with depressive symptoms. Literate study participants had a protective effect against depressive symptoms, as literate study participants were 76% less likely to have depressive symptoms than their illiterate counterparts, which aligns with studies conducted in India [25] and Sudan [26]. The social causation perspective suggests that higher education can lead to better socioeconomic conditions, reduced stress, improved coping strategies, and a decreased risk of depressive symptoms [35]. In addition, higher education levels have a long-term effect on depressive symptoms and can help reduce the risk of depression later in life [36]. However, previous studies conducted in Nepal [12], Pakistan [37], and Bhutan [22] did not find any association between education and depressive symptoms.

PLHIV in the poorest wealth quintile were more likely to have depressive symptoms than in the wealthiest quintile. The findings of this study are supported by a study conducted in India [38], Vietnam [19], and Cameroon [39], where PLHIV with low socioeconomic status were found to have more depressive symptoms compared to PLHIV with high socioeconomic status. Socioeconomic status can impact the development of mental illness through adverse, economically stressful conditions among lower-income groups [40]. Also, having financial resources can prevent the stressors that drive depression and reduce the blow of disruptions in daily living [40].

PLHIV with a period of ART initiation ≤12 months were more likely to have depressive symptoms than those with a period of ART initiation >12 months, supported by a study conducted in Nepal [13] and Vietnam [19]. PLHIV with a shorter duration of ART initiation might feel burdened with taking medicine, maintaining an ART adherence rate, and having less confidence regarding the positive effects of ART drugs which might trigger stress levels and develop depressive symptoms. With longer ART initiation duration and experiencing the benefits of ART drugs, HIV patients may feel more normal regarding their disease status, have lower stress levels, and have decreased depressive symptoms. However, previous studies conducted in China, Bhutan, Ethiopia, and Cameroon [9, 22, 32, 39] did not show any association. This could be because of the different cutoff points used for the duration of ART initiation.

The CD4 count of PLHIV was significantly associated with depressive symptoms. PLHIV with a CD4 count <200 cells/mm^3^ was 2.61 times more likely to have depressive symptoms than those with HIV with a CD4 count ≥200 cells/mm^3^. This study's findings are in alignment with the other studies conducted in Pakistan [24], Vietnam [33], Ethiopia [41], Cameroon [21, 39, 42], and Nigeria [43]. A lower CD4 count often results in an AIDS-defining illness and increased mortality [44]. HIV patients with low CD4 cell counts might have an increased fear of dying, leading to depressive symptoms. Also, the inability to maintain a normal CD4 cells count despite being on ART medication might discourage HIV patients and help with the progression of depressive symptoms [21].

To the best of our knowledge, this is the first global study to assess the association between the time gap between HIV diagnosis and ART initiation and depressive symptoms among PLHIV. Our findings suggest that those diagnosed with HIV and who began ART within three months were 50% less likely to experience depressive symptoms than those who waited longer. People who have been newly diagnosed with HIV can be considered to have stressful life events [45] and have increased concern about their long-term health, social stigma, and medication [46], all of which may result in stress, psychological burden, and depressive symptoms. Reducing the time gap between HIV diagnosis by the timely initiation of ART medication and counseling services may help newly diagnosed individuals build confidence, develop coping strategies, and reduce stress levels, burden, and depressive symptoms associated with HIV infection.

Though Nepal has made good progress in its HIV prevention and control program, policies addressing the mental health needs of PLHIV are still lacking. Regardless of the significant predictors found in this study, there is a need to develop HIV care guidelines regarding the management of depressive symptoms in PLHIV. Interventions, including cognitive-behavioral group plans and community-based interpersonal psychotherapy can be beneficial in reducing depressive symptoms in PLHIV, especially in developing countries like Nepal [47, 48]. Having family, friends, and special someone can act as constant support physically, mentally, and emotionally in particular matters related to taking care of the health needs of PLHIV, thus improving overall well-being and quality of life among PLHIV [49]. ART centers act as focal points for providing treatment, care, and support for PLHIV; hence, staff working in ART centers need to be trained regarding the assessment of determinants of mental health, social needs, and clinical issues of PLHIV. Family members and friends need to be involved from the beginning in providing care and support to PLHIV through proper counseling services and peer support groups. The role of the private and developmental sectors can be explored in addressing the mental health needs of PLHIV.

## 5. Conclusion

More than one-fourth (26.8%) of the people living with HIV had depressive symptoms. Educational status, wealth quintile, CD4 count, duration of ART initiation, and time difference between HIV diagnosis and ART initiation were found to be significant predictors of depressive symptoms among PLHIV. Routine mental health screening for all PLHIV attending ART centers should be done to identify the risk of depressive symptoms. Also, mental health care services need to be integrated into national HIV prevention and control programs. The PLHIV group should be made aware of the importance of timely treatment initiation and adherence to ART. An enabling environment should be created to facilitate the rapid enrollment of individuals newly diagnosed with HIV for ART services, thereby reducing the time gap between HIV diagnosis and ART initiation. There is a need to advocate for making policies that prioritize mental health services within HIV care and allocate resources accordingly to address the mental health needs of PLHIV. More studies regarding mental health issues among PLHIV need to be conducted, which can provide evidence to other researcher's health care service providers and policymakers for designing effective interventional programs to address the issue. Since most of the studies conducted are cross-sectional in nature, there is a need to conduct analytical studies which will help establish causal relationships.

## Figures and Tables

**Table 1:** Sociodemographic characteristics of study participants at selected ART centers in Lumbini province, 2020.

Variables (*n* = 406)	Category	Frequency	Percentage
Age of respondent	≤40	200	49.3
	>40	206	50.7

Sex	Male	199	49
	Female	207	51

Ethnicity	Dalit	58	14.3
	Janjati	108	26.6
	Religious minorities	122	30
	Upper caste	118	29.1

Educational status	Literate	121	29.8
	Illiterate	285	70.2

Occupational status	Unemployed	82	20.2
	Agriculture	131	32.3
	Labor/wage worker	70	17.2
	Self/employed	123	30.3

Family type	Nuclear	247	60.8
	Joint	159	39.2

Tobacco products consumption	Yes	111	27.3
	No	295	72.7

Alcohol consumption	Yes	108	26.6
	No	298	73.4

Marital status	Married	258	63.5
	Unmarried/widow/separated	148	36.5

**Table 2:** Sociodemographic characteristics of the respondent's partner at selected ART centers in Lumbini province, 2020.

Variables (*n* = 258)	Category	Frequency	Percentage
Age	≤40	148	57.4
	>40	110	42.6

Educational status	Literate	79	30.6
	Illiterate	179	69.4

Occupational status	Unemployed	64	24.8
	Agriculture	90	34.9
	Labor/wage worker	37	14.3
	Self/employed	67	26.0

Tobacco products consumption	Yes	62	24.0
	No	196	76.0

Alcohol consumption	Yes	62	24.0
	No	196	76.0

**Table 3:** Depression and clinical characteristics of study participants at selected ART centers in Lumbini province, 2020.

Variables (*n* = 406)	Category	Frequency	Percentage
Depression	Yes	109	26.8
	No	297	73.2

Period of diagnosis	≤12 months	62	15.3
	>12 months	344	84.7

Period of ART initiation	≤12 months	65	16
	>12 months	341	84

Time difference between diagnosis and ART initiation	≤3 months	316	77.8
	>3 months	90	22.2

WHO clinical stage	Stage I	205	50.5
	Stage II	78	19.2
	Stage III	98	24.1
	Stage IV	25	6.2

CD4 count (cells/mm^3^)	<200	105	25.9
	≥200	301	74.1

**Table 4:** Sociodemographic predictors of depression at selected ART centers in Lumbini province, 2020.

Variables	Depression		COR	95% CI	AOR	95% CI
	Yes (%)	No (%)				
*Age of respondent*						
≤40	48 (24.0)	152 (76.0)	1.08	0.96–1.21		
>40	61 (29.6)	145 (70.4)	1			

*Sex*						
Male	55 (27.6)	144 (72.4)	1.08	0.69–1.67		
Female	54 (26.1)	153 (73.9)	1			

*Ethnicity*						
Dalit	16 (27.6)	42 (72.4)	0.98	0.48–1.98		
Janjati	20 (18.5)	88 (81.5)	0.58	0.31–1.10		
Religious minorities	40 (32.8)	82 (67.2)	1.25	0.72–2.18		
Upper	33 (28.0)	85 (72.0)	1			

*Educational status*						
Literate	44 (36.4)	77 (63.6)	0.82	0.71–0.95^*∗*^	0.24	0.10–0.61^*∗∗*^
Illiterate	65 (22.8)	220 (77.2)	1		1	

*Occupational status*						
Unemployed	27 (32.9)	55 (67.1)	3.28	1.63–6.60^*∗*^	1.51	0.51–4.42
Agriculture	39 (29.8)	92 (70.2)	2.83	1.48–5.40^*∗*^	1.63	0.60–4.37
Labor/wage worker	27 (38.6)	43 (61.4)	4.19	2.05–8.56^*∗*^	1.17	0.39–3.50
Self/employed	16 (13.0)	107 (87.0)	1		1	

*Family type*						
Nuclear	53 (21.5)	194 (78.5)	0.50	0.32–0.78^*∗*^	0.99	0.49–2.00
Joint	56 (35.2)	103 (64.8)	1		1	

*Tobacco consumption*						
Yes	29 (26.1)	82 (73.9)	0.95	0.57–1.55		
No	80 (27.1)	215 (72.9)	1			

*Alcohol consumption*						
Yes	33 (30.6)	75 (69.4)	1.28	0.79–2.08		
No	76 (25.5)	222 (74.5)	1			

*Marital status*						
Married	62 (24.0)	196 (76.0)	0.68	0.43–1.06		
Unmarried/widow/separated	47 (31.8)	101 (68.2)	1			

*Wealth quintile*						
Poorest	52 (64.2)	29 (35.8)	9.37	4.44–19.80^*∗*^	7.28	2.22–23.87^*∗∗*^
Poorer	23 (28.4)	58 (71.6)	2.07	0.96–4.45	2.23	0.77–6.47
Middle	11 (13.6)	70 (86.4)	0.82	0.34–1.96	0.82	0.29–2.32
Richer	10 (12.2)	72 (87.8)	0.72	0.29–1.76	0.65	0.21–1.98
Richest	13 (16.0)	68 (84.0)	1		1	

*Age of respondent's partner*						
≤40	34 (23.0)	114 (77.0)	1.03	0.89–1.18		
>40	28 (25.5)	82 (74.5)	1			

*Educational status of respondent's partner*						
Literate	24 (30.4)	55 (69.6)	1.61	0.89–2.94		
Illiterate	38 (21.2)	141 (78.8)	1			

*Occupational status of respondent's partner*						
Unemployed	22 (34.4)	42 (65.6)	3.37	1.41–8.06^*∗*^	2.05	0.76–5.53
Agriculture	22 (24.4)	68 (75.6)	2.08	0.89–4.88	1.32	0.45–3.91
Labor/wage worker	9 (24.3)	28 (75.7)	2.07	0.74–5.79	1.39	0.43–4.48
Self/employed	9 (13.4)	58 (86.6)	1		1	

*Tobacco consumption by respondent's partner*						
Yes	10 (16.1)	52 (83.9)	0.53	0.25–1.12		
No	52 (26.5)	144 (73.5)	1			

*Tobacco consumption by respondent's partner*						
Yes	14 (22.6)	48 (77.4)	0.89	0.45–1.77		
No	48 (24.5)	148 (75.5)	1			

^*∗*^Variables entered to multivariate regression model. ^*∗∗*^Statistically significant at *p* value <0.05, adjusted model.

**Table 5:** Clinical predictors of depression at selected ART centers in Lumbini province, 2020.

Variables	Depression		COR	95% CI	AOR	95% CI
	Yes	No				
*Period of diagnosis*						
≤12 months	21 (33.9)	41 (66.1)	1.49	0.83–2.65		
>12 months	88 (25.6)	256 (74.4)	1			

*Period of ART initiation*						
≤12 months	24 (36.9)	41 (63.1)	1.76	1.00–3.08^*∗*^	1.88	1.03–3.42^*∗∗*^
>12 months	85 (24.9)	256 (75.1)	1		1	

*Time difference between period of diagnosis and ART initiation*						
≤3 months	77 (24.4)	239 (75.6)	0.58	0.35–0.96^*∗*^	0.50	0.29–0.86^*∗∗*^
>3 months	32 (35.6)	58 (64.4)	1		1	

*WHO clinical stage*						
Stage I	48 (23.4)	157 (76.6)	0.78	0.31–1.99		
Stage II	22 (28.2)	56 (71.8)	1.01	0.37–2.75		
Stage III	32 (32.7)	66 (67.3)	1.24	0.47–3.28		
Stage IV	7 (28.0)	18 (72.0)	1			

*CD4 count*						
<200	44 (41.9)	61 (58.1)	2.61	1.62–4.21^*∗*^	2.50	1.54–4.06^*∗∗*^
≥200	65 (21.6)	236 (78.4)	1		1	

^*∗*^Variables entered to multivariate regression model. ^*∗∗*^Statistically significant at *p* value <0.05, adjusted model.

## Data Availability

The datasets used and/or analyzed during the current study are available from the corresponding author on reasonable request.

## Abbreviations

AOR: Adjusted odds ratio
ART: Antiretroviral therapy
CD4: Cell differentiation
CI: Confidence interval
COR: Crude odds ratio
HIV: Human immunodeficiency virus
PLHIV: People living with HIV
WHO: World Health Organization.
